# Design and Validation of a Breathing Detection System for Scuba Divers

**DOI:** 10.3390/s17061349

**Published:** 2017-06-09

**Authors:** Corentin Altepe, S. Murat Egi, Tamer Ozyigit, D. Ruzgar Sinoplu, Alessandro Marroni, Paola Pierleoni

**Affiliations:** 1 Bogazici Underwater Research Center, Yavuzturk Sk. 32/1 Altiyol, 34716 Istanbul, Turkey; deniz@innovasub.com; 2 Information Engineering Department, Marche Polytechnic University, Via Brecce Bianche 12, 60131 Ancona, Italy; p.pierleoni@univpm.it; 3 Computer Engineering Department, Galatasaray University, Ciragan Cad. 36 Ortakoy, 34349 Istanbul, Turkey; megi@gsu.edu.tr (S.M.E.); tamerozyigit@gmail.com (T.O.); 4 DAN Europe Research Division, Contada Padune 11, 64026 Roseto degli Abruzzi, Italy; amarroni@daneurope.org

**Keywords:** breathing monitor, inhalation detection, two-stage regulator, continuous monitoring, diving, drowning, hyperventilation, hypercapnia, skip-breathing

## Abstract

Drowning is the major cause of death in self-contained underwater breathing apparatus (SCUBA) diving. This study proposes an embedded system with a live and light-weight algorithm which detects the breathing of divers through the analysis of the intermediate pressure (IP) signal of the SCUBA regulator. A system composed mainly of two pressure sensors and a low-power microcontroller was designed and programmed to record the pressure sensors signals and provide alarms in absence of breathing. An algorithm was developed to analyze the signals and identify inhalation events of the diver. A waterproof case was built to accommodate the system and was tested up to a depth of 25 m in a pressure chamber. To validate the system in the real environment, a series of dives with two different types of workload requiring different ranges of breathing frequencies were planned. Eight professional SCUBA divers volunteered to dive with the system to collect their IP data in order to participate to validation trials. The subjects underwent two dives, each of 52 min on average and a maximum depth of 7 m. The algorithm was optimized for the collected dataset and proved a sensitivity of inhalation detection of 97.5% and a total number of 275 false positives (FP) over a total recording time of 13.9 h. The detection algorithm presents a maximum delay of 5.2 s and requires only 800 bytes of random-access memory (RAM). The results were compared against the analysis of video records of the dives by two blinded observers and proved a sensitivity of 97.6% on the data set. The design includes a buzzer to provide audible alarms to accompanying dive buddies which will be triggered in case of degraded health conditions such as near drowning (absence of breathing), hyperventilation (breathing frequency too high) and skip-breathing (breathing frequency too low) measured by the improper breathing frequency. The system also measures the IP at rest before the dive and indicates with flashing light-emitting diodes and audible alarm the regulator malfunctions due to high or low IP that may cause fatal accidents during the dive by preventing natural breathing. It is also planned to relay the alarm signal to underwater and surface rescue authorities by means of acoustic communication.

## 1. Introduction

SCUBA diving tourism has grown to become a multibillion dollar industry, drawing as many as 6 million active SCUBA divers worldwide [1]. One of the major concerns of the diving industry is to secure a significant and sustained reduction in the number of fatal and major accidents in activities that may jeopardize human lives. Divers Alert Network (DAN) has reported 146 recreational diving fatalities in 2014 world-wide, excluding breath-holding fatalities [2]. Of the 44 cases for which the cause of death was determined, 21 (48%) were caused by drowning, 3 (7%) were probable drownings, 7 (16%) were cardiac event, and 2 (5%) probable cardiac events. Similar proportions were reported over the period of 2010–2013 with 53% drownings, and 28% cardiovascular diseases [3]. It is a reasonable assumption that a proportion of these fatalities could have been avoided if a continuous monitoring of the breathing of the divers had been in place and had reported the emergency when identified that the diver stopped breathing.

The U.S. Navy has identified breathing related problems that may lead to accidents, such as drowning, threatening the lives of SCUBA divers [4]. It is reported that novice divers are likely to breathe deeper and more frequently, causing to deplete the gas supply faster than planned, which may cause the diver to drown. However, it is recommended against skip-breathing, which occurs when a long unnatural pause is inserted between each breath, as it leads to hypercapnia—an abnormally high level of carbon dioxide in the blood and body tissues-unconsciousness, and death. Involuntary hyperventilation—breathing more than is necessary to keep the body’s carbon dioxide tensions at proper level—can be triggered by fear experienced during stressful situations, the anxiety of the first few dives or the discomfort caused by the SCUBA equipment, the increase in static lung loading, or the increase in breathing resistance. A device monitoring the frequency of breathing can obviously save lives while preventing drowning accidents, injuries and fatalities related to hyperventilation and skip-breathing. In addition to recreational diving, the Diving Medical Advisory Committee has recommended monitoring the breathing of commercial divers to improve safety as early as 1979 [5]. Despite the health concerns of recreational and commercial divers, there are a limited number of health monitoring systems for SCUBA diving due to the nature of challenging environment. These efforts are mainly committed to monitoring the electrocardiogram (ECG) [6,7,8,9,10], blood glucose [11], and heart rate [12]. There is one commercially available dive computer which records the heart rate (Galileo Sol, Scubapro, El Cajon, CA, USA). Moreover, these monitoring attempts have never addressed breathing detection which can prevent major cause of deaths (drowning) and SCUBA diving injuries. Methods have been developed to detect and locate SCUBA divers remotely using passive and active acoustic monitoring for security as well as safety applications [13,14,15,16], using the sound emitted by the breathing of the diver and its regularity. There are, however, no studies on such systems applied to a group of several divers.

While there are numerous methods of pattern recognition and classification of time-varying signals applied widely to all fields of research in medicine, engineering or computer science [17,18,19], the vast majority of the solutions in the literature make use of algorithms requiring intensive computation, which would not be able to run on small, embedded systems with limited resources.

This study introduces the design and validation of a simple and robust breathing detection method based on identification of the sudden pressure drops in the hose of the breathing regulator of the diver triggered by inhalation.

## 2. Materials and Methods

### 2.1. Subjects

Eight scuba divers, 7 males and 1 female, volunteered for this study. They were all professional divers. There were two divers with Advanced level certification, four with Dive Master and two with Instructor level. They averaged an experience of 1300 dives per diver, and 226 dives in the past year. The age averaged 30.5 years (standard deviation (SD) of 7.3 years), ranging from 23 to 46 years old. 5 were smokers and 4 practiced a physical activity regularly. All divers had an up-to-date medical fitness-to-dive certificate delivered by a diving medical specialist. No medical condition was declared. Of the 7 male subjects, the weight averaged 73.3 kg (SD: 8.7 kg) and the height averaged 176.7 cm (SD: 5.2 cm). Written informed consent was obtained from all participants after a full explanation of the aims and procedures.

### 2.2. Recording System

The central piece of equipment of a scuba is the regulator, which drops the compressed gas contained in the tank to a breatheable pressure for the diver. This regulator is built on two stages: a first stage drops the pressure from the tank generally rated to 200 bar to an IP and the second stage drops the IP to the ambient pressure (PB). In the modern regulators, the IP is set to an absolute pressure of PB+9.6 bar while the diver is not breathing. When the diver starts breathing, there is a sudden decrease in the IP value which returns gradually to its nominal value (PB+9.6 bar). The authors postulated that the detection of this sudden decrease triggered by inhalation can be used to detect respiratory frequency and can be used to identify the absence of breathing that will lead to drowning or anomalies such as hyperventilation and skip-breathing. A device was designed to detect the IP pressure change when mounted to the pressure hose of the buoyancy compensator device (BC) of the diver.

**Figure 1:**
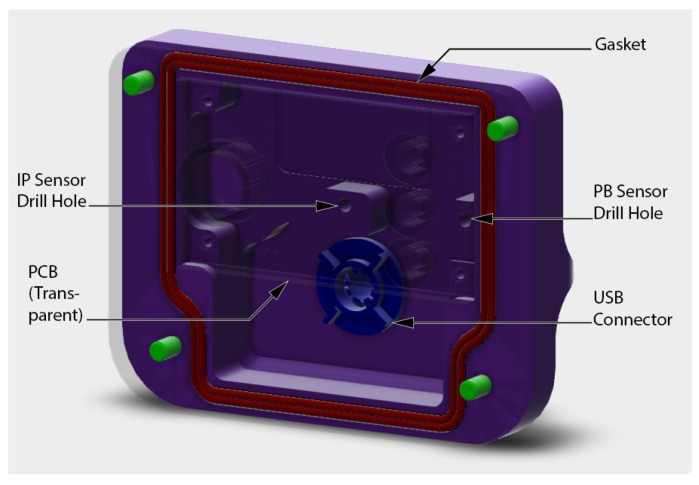
View of the 3D design of the device’s casing.

**Figure 2:**
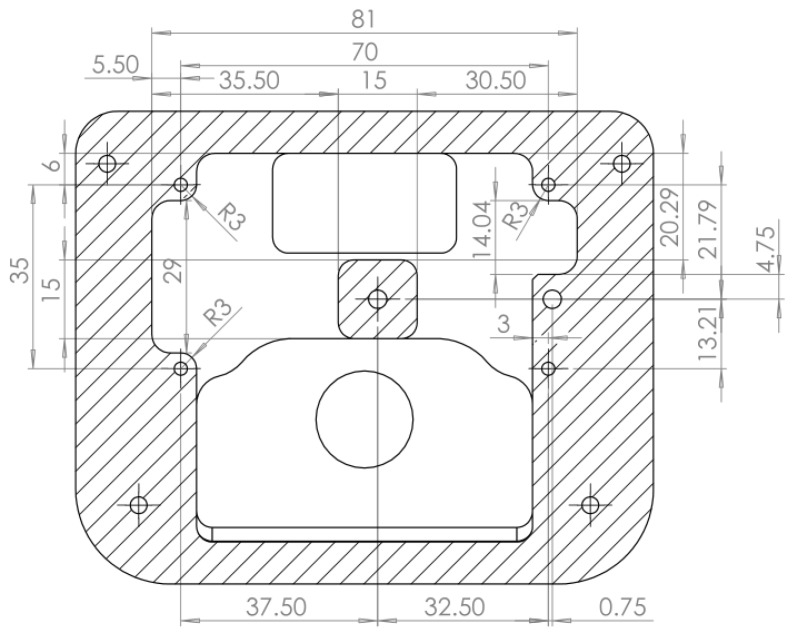
Technical drawing of the device’s casing, dimensions in mm.

A system enabling recording of IP and PB signals designed in a previous study [20] was used for the experimental procedure. It is an electronic device built around a microcontroller (MSP430F5529, Texas Instruments Incorporated, Dallas, TX, USA), two pressure sensors (MS5837, TE Connectivity, Schaffhausen, Switzerland) and an internal memory component. Its firmware was developed in C language with Code Composer Studio 6.0 IDE (Texas Instruments Incorporated, Dallas, TX, USA). A custom made mechanical housing was designed to accommodate the device and was manufactured in aluminum with a computer numerical control router (CNC) machine. A view of the 3D design is given in Figure 1 and the technical drawing of the part is displayed in Figure 2. The design uses standard BC “quick-disconnect” type male and female connectors for an easy mounting on SCUBA equipment without requiring any additional adapter. The IP and PB sensors and well as the light-emitting diodes are to be assembled on the same electronic board for an efficient mounting when producing the device. A gasket ensures sealing of the device for depths tested up to 25 m. 

**Figure 3:**
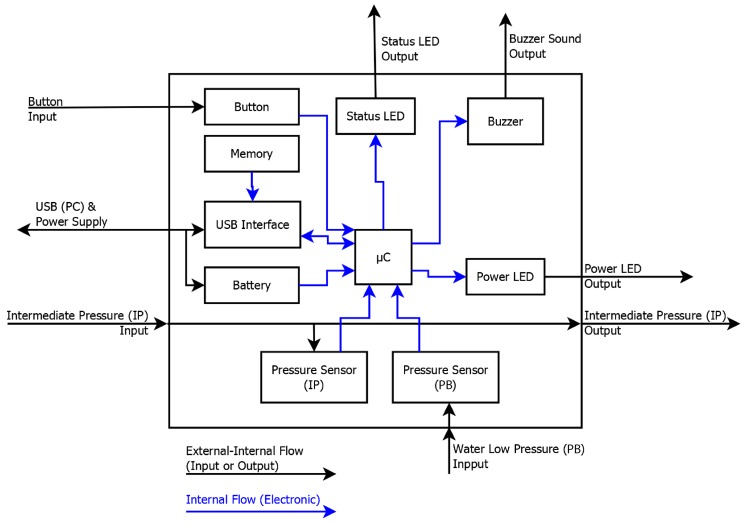
Block diagram of the recording system’s composition.

**Figure 4:**
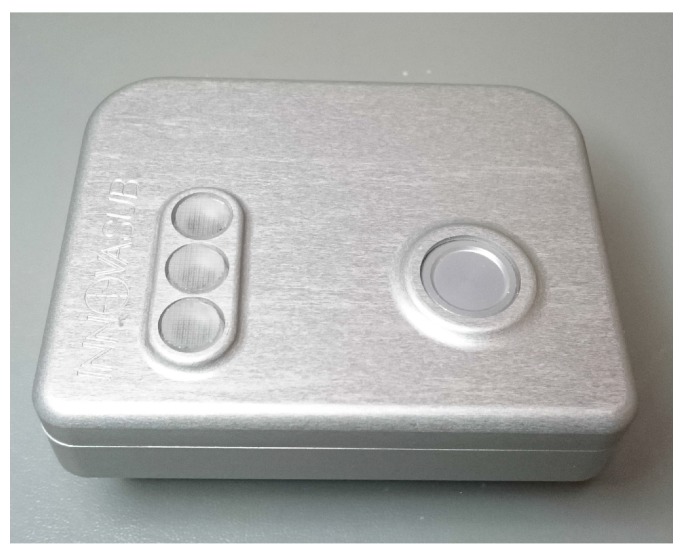
Photograph of the recording system.

**Figure 5:**
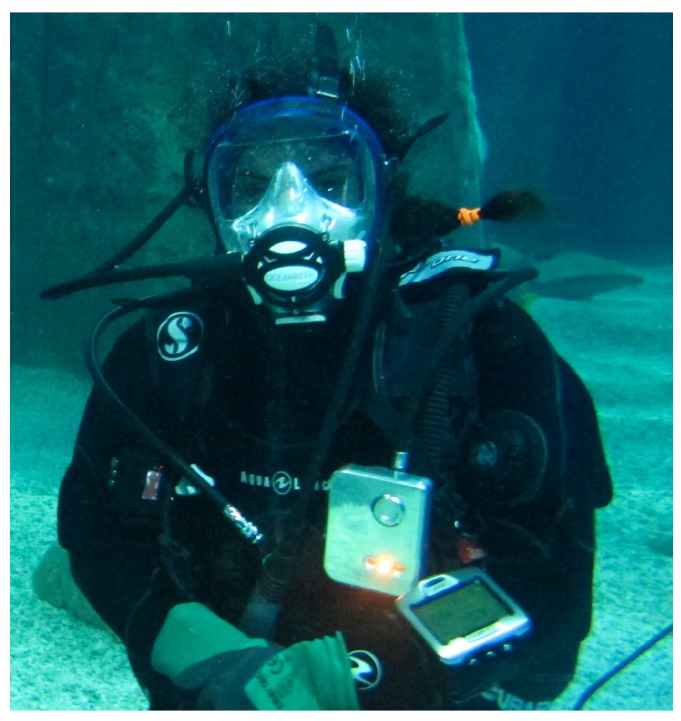
Photograph of a diver equipped with the recording system (above left arm) and a video camera (above right arm).

This system is plugged to the IP hose of the BC which is normally used to inflate the BC, to sense the IP. Figure 3 details the components of the system and their communication flow in a block diagram. Figure 4 is a photograph of the system used for the experiment. Figure 5 shows the system in use underwater.

The system senses the ambient pressure of the air with its PB sensor at start up, which is later used to calculate the depth of the diver while underwater. It starts recording in memory the PB and IP signals as soon as the diver’s depth reaches 0.5 m. The record ends after 5 min have been spent at a depth shallower than 0.3 m. Specifications of the pressure sensors and recording resolution of IP and PB signals are given in Table 1. All recorded data is transferred to a personal computer (PC) application after the dive through a USB connection.

### 2.3. Detection Algorithm

A detection algorithm was designed to fit the following constraints: it should enable (1) a live detection of inhalation events with (2) a maximum delay of ten seconds and be (3) light-weight in order to be embedded in a non digital signal processing (DSP), ultra-low power microcontroller with a RAM limited to 6 kBytes for the algorithm and a processor frequency of maximum 25 MHz, such the MSP430F5529 or equivalent. The algorithm was designed to be light-weight in order to enable embedding it in not only a custom designed electronics, but also in common existing dive equipment electronics such as dive computers. Modern dive computers use power-efficient microcontrollers such as the MSP430F5529 to perform multiple dives with a single coin cell battery.

The algorithm proposes to detect inhalation events by the diver and to report the time of each detected inhalation, which by extent corresponds to monitoring the breathing of the diver.

**Figure 6:**
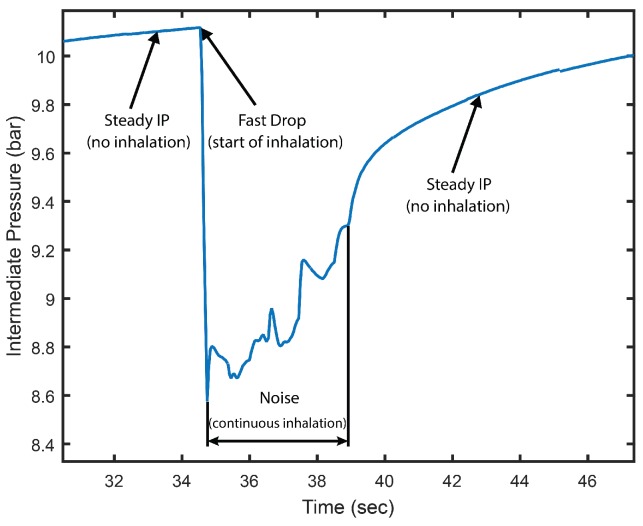
Intermediate pressure (IP) signal on an inhalation event.

Figure 6 details the IP signal recorded on a single inhalation event, which the algorithm aims at identifying. The algorithm proceeds in four steps. The first step subtracts the PB signal from the IP signal, applying Equation (1).

(1) $$IP_{C}=IP-PB$$

**Figure 7:**
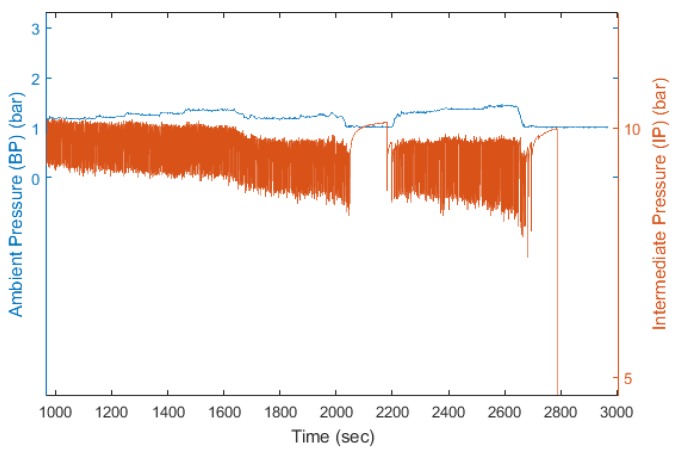
Extract of ambient pressure (PB) and IP signal of dive 3.

This is to compensate the fact that the IP is 9.6 bar above the PB in scuba regulators, as it is illustrated in Figure 7 where the correlation between PB and IP can be noticed visually. Therefore, the IP is affected by an offset directly affected by the depth of the diver. The first step aims at removing the depth variations from the IP signal. It must be noted that since the PB signal is sampled at 1 Hz and the IP signal is sampled at 20 Hz, each sample of the PB signal was repeated 20 times to match with the IP signal frequency, making the PB a step signal.

**Figure 8:**
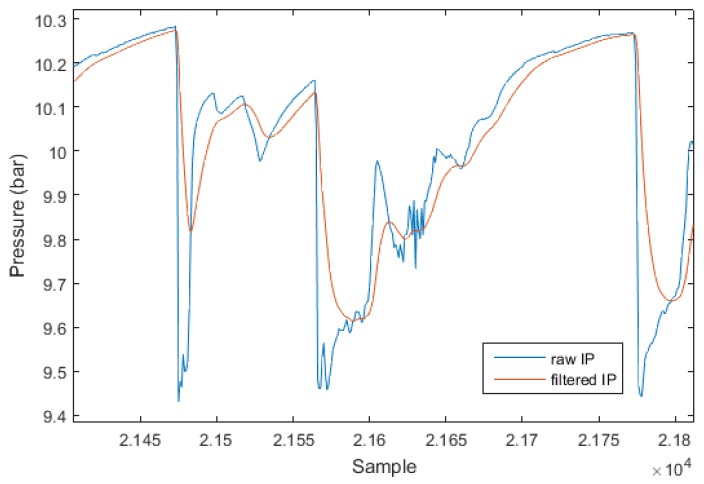
Extract of dive 16 IP unfiltered signal (**blue**) and signal filtered with first-order, low-pass Butterworth filter with normalized cut-off frequency of 0.37 and delay compensated (**red**).

In the second step, the obtained signal is filtered with a first order, low-pass, Butterworth filter with a normalized cut-off frequency Fc to remove the noise of the pressure sensor and the regulator itself during continuous inhalation. Figure 8 shows the effect of the low-pass filter on an extract of IP signal from dive 16.

In the third step, a window of the first *M* samples of the obtained signal is analyzed, where *M* is a positive integer. The minimum and maximum pressures on this *M*-window are determined, and a Threshold variable is defined as:

(2) ∀Tr∈[0,1],Threshold=Tr.(Max-Min)+Min

where Max is the maximum pressure on the *M*-window, Min is the minimum pressure on the *M*-window and Tr is an arbitrary real number between 0 and 1.0. Threshold is expressed in bar.

**Figure 9:**
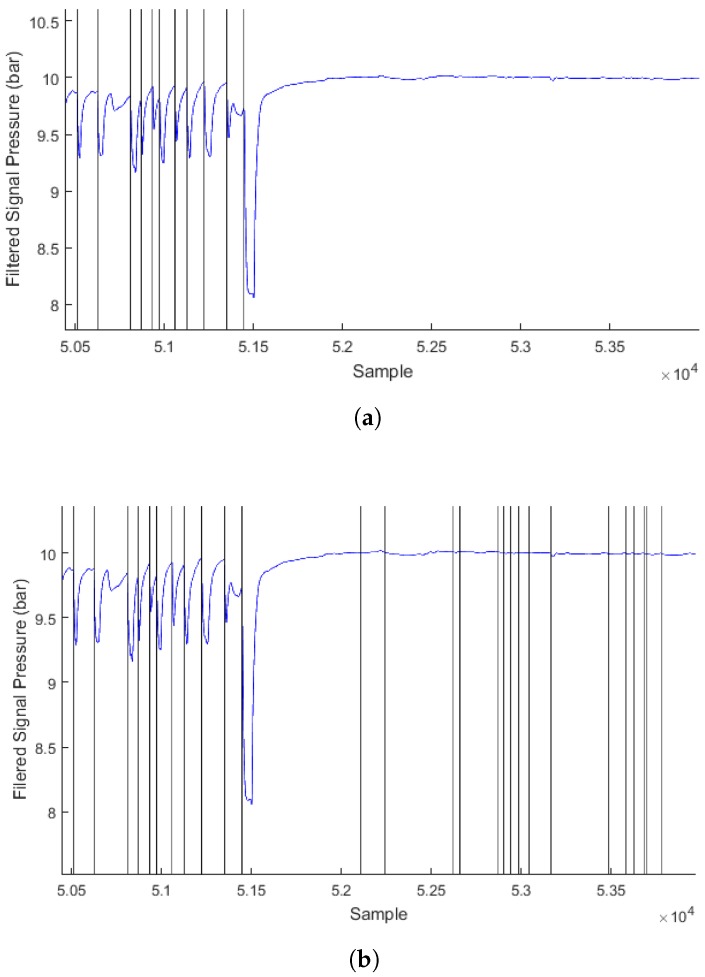
Extract of dive 16 filtered signal (blue) and inhalation events detected by the algorithm (vertical black lines), with M=200, N=100, Tr=0.55 and Fc=0.037—Diff=0.3 bar (**a**) and Diff=0.0 bar (**b**).

In the fourth step, the algorithm counts every time the signal sample value goes from above the threshold value to below or equal the threshold value within the window, such that

(3) $$\left\{\begin{array}{cc} S[n] & >Threshold\\ \mathrm{and}\\ S[n+1] & \leq Threshold \end{array}\right.$$

where *n* is the sample number in the analyzed signal and S[n] is the pressure value of the $n^{th}$ sample, in bar. Such condition is considered as an inhalation event and the sample number *n* is reported. The algorithm then moves the window by *N* samples forward (with N<M) and repeats from the third step until the whole signal is analyzed, with the difference that in the fourth step the inhalation events are detected in a window of only the *N* latest samples in the *M* window, in order to avoid counting an event twice (since N<M). Additionally, no inhalation event is accounted in the fourth step if the value (Max-Min) is smaller than a defined, arbitrary value named Diff. This is to avoid noise being identified as an event when no actual inhalation occurs, and the IP signal stabilizes at an asymptotic value. This effect is illustrated in Figure 9 where Diff was set to a value of 0.3 bar on the top figure—it is observed that no inhalation is detected when the diver stops breathing after sample 52000—and Diff was set to 0.0 bar on the bottom figure, where inhalation events are wrongly detected after the diver stops breathing.

To sum-up, the detection algorithm is governed by the following variables: *M* unitless, *N* unitless, Fc unitless, Tr unitless, and Diff in bar.

### 2.4. Experimental Procedure

Inhalation events were assessed simultaneously by the PB and IP recording system used in conjunction with the detection algorithm, and an underwater video camera (Edge X, Intova, Honolulu, HI, USA), at the aquarium Florya of Istanbul, Turkey, during the period of 24 October to 25 November 2016. Two tanks were used for the experiment. Tank 1 has a water temperature of 21.5 °C, a maximum depth of 7 m and hosts various species of sharks, leerfish, epinephelus marginatus, temperate basses, rays and other various marine life. Tank 2 has a temperature of 18 °C and a maximum depth of 6 m. Tank 2 hosts two banded breams, catfish, and gilt-head breams. The aquarium requires 13 dives per week for the maintenance of these tanks, involving sand and window cleaning.

Each subject was equipped with a scuba diving dry suit for thermal isolation. They equipped the recording system above their left arm, plugged between the first stage regulator hose and the BC connector which is the jacket enabling divers to balance their buoyancy underwater. Each participant was also equipped with a recording video camera fixed near their right shoulder prior to each test. Figure 5 shows the configuration of the device equipping a diver. Two dives were sampled per diver, with a surface interval of 20 min to 7 days between the two dives.

Each dive lasted between 49 and 69 min, and were performed at a maximum depth of 6.8 m. All tests were followed and data collected by one blinded observer who was not aware of the results obtained during the tests. The data was analyzed and inhalation events counted by another blinded observer, after all dives were performed and all data collected.

The first of the two dives involved the tasks of cleaning the glass of the aquarium. This was reported before the start of the experiment as a task demanding physical effort and constant movement. On the second dive, the subjects performed the siphoning of the sand in the aquarium, which consists in cleaning the sand. This activity was reported before the start of the experiment as less demanding physically and requiring less movement.

Participants were instructed to perform their work activities as usual. The assignment of the tank for the dive was not chosen by the experiment. The divers were instructed to follow at all times the safety protocol they were trained to, and to abandon the test should any equipment fail and cause to threaten their safety.

The camera started recording prior to the descent in the water and was stopped after the diver reached the surface at the end of the dive. The video record was backed up in a PC after the dive. The PB and IP recording system was started prior to the descent in the water. The system was configured to start recording PB and IP signals as soon as the diver’s depth reaches 0.5 m or deeper. The system stopped the record after 5 min have been spent on the surface. The data were backed up to a PC after the dive.

### 2.5. Performance Analysis

All analyses were performed with the numerical computing software Matlab 2015a for Windows (The MathWorks, Inc., Natick, MA, USA). Each inhalation event in the collected IP signals was manually marked by a blinded observer by analyzing only the graph of the unfiltered IP signal. The inhalation detection algorithm was implemented in Matlab scripts and true positives (TP), FP and false negatives (FN) events were counted for each recorded dive. Similarly to spike detection in EEG signal analysis described in [21], it is unclear what a true negative (TN) event is in the case of inhalation detection analysis and it will be therefore ignored.

**Figure 10:**
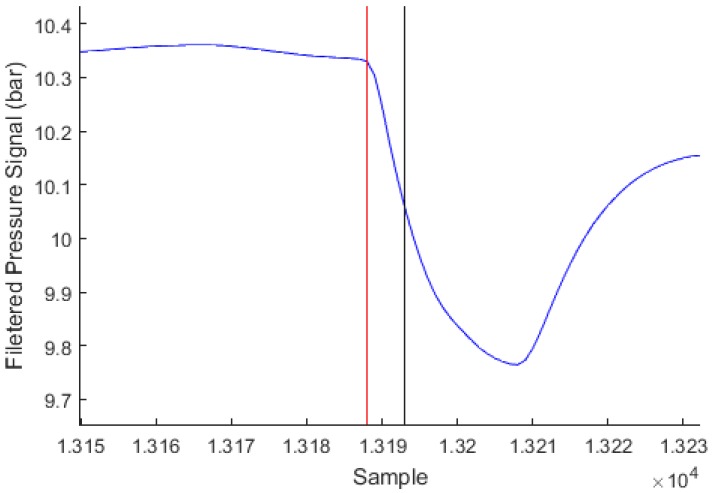
Extract of dive 16 filtered signal with marked inhalation time (**red**) and inhalation time as detected by the algorithm (**black**).

Each inhalation event detected by the algorithm is marked by its sample number *n* in the IP signal. It is considered a TP if a manually marked event is present within the range [n-15;n+5]. It is considered a FP otherwise. For each manually marked event with a sample number m, it is considered a FN if no inhalation event is detected by the algorithm within a range of [m-5;m+15]. While the observer generally marks the event at the very beginning of the pressure drop, the algorithm is designed to mark the event at about half the way during the pressure drop, approximately 500 ms after the start of the drop as shown in Figure 10. Therefore, the asymmetric ranges of detection account for the fact that the observer-marked events are generally placed 500 ms before the algorithm-detected events, and this is considered acceptable.

The total sensitivity was computed for the whole dataset as:

(4) $$Sensitivity=\frac{TP}{TP+FN}$$

where TP is the total number of events correctly detected by the algorithm on the 16 dives, and FN is the total number of missed (false negative) events on the 16 dives. The ratio *R* is defined as:

(5) $$R=\frac{FP}{TP+FN}$$

where FP is the total number of falsely detected events by the algorithm on the 16 dives. It must be noted that specificity cannot be quantified as TN was not defined.

### 2.6. Optimization

An optimization criteria was defined as

(6) Criteria=(1−Sensitivity)+2·R

over the whole dataset. More weight was given to the ratio *R* than the sensitivity of the algorithm, as the end goal was to detect when the diver is not breathing, defining a case of emergency. A high ratio *R* expresses an algorithm with many FP, meaning that an inhalation event might be detected when there is actually none, which by extension may lead to believe the diver is breathing when he actually isn’t.

**Figure 11:**
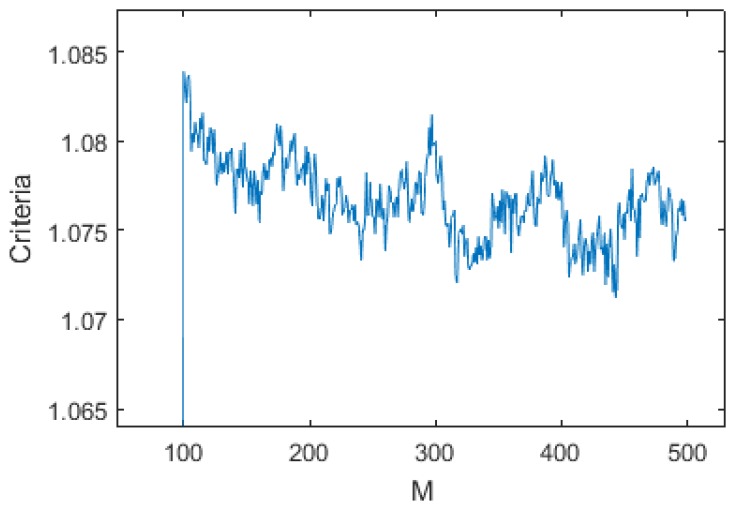
Criteria vs. M over the 16 dives data with N=100, Fc=0.037, Tr=0.55 and Diff=0.3bar.

**Figure 12:**
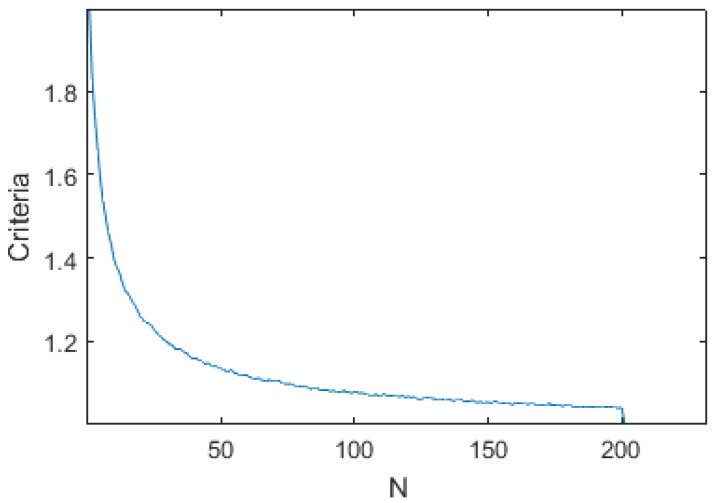
Criteria vs. N over the 16 dives data with M=200, Fc=0.037, Tr=0.55 and Diff=0.3bar.

An initial study has shown that *M* and *N* had little influence on the global performance of the algorithm, as shown in Figure 11 and Figure 12. They were therefore fixed to M=200 (10 s) and N=100 (5 s).

The constants of the algorithm Tr, Fc and Diff were then optimized with Matlab to minimize the output Criteria for the given dataset.

### 2.7. Validation

Two operators were given the video records of the dives and were instructed to mark down the time of each inhalation based on the sound and, when available, the vision of the diver’s face and bubbles, using the software SubtitleEdit (Nikolaj Lynge Olsson, Copenhagen, Denmark). They marked down the 10 first min of each dive. The operators were blinded observers with no access to the PB and IP data recorded by the system, and no access to one another’s analysis.

The vectors of events marked by the observer 2 were compared to the marked event vectors of observer 1 in order to evaluate the reproducibility of the marking process. The criteria of performance used for this comparison was the same as the criteria used to evaluate the performance of the algorithm: an event marked by observer 1 is considered a TP if an event marked by observer 2 is present within the range [n-15;n+5]. It is considered a FP otherwise. Similarly, a FN is counted if no inhalation event of observer 2 is present within a range of [m-5;m+15]. The ranges being asymmetric, the events of observer 1 were then compared against the events of observer 2.

For each dive, the events marked by the blinded observers were then synchronized with the recording system’s detected event vectors. The video camera started the video recording before the start of a dive, a time offset was applied to the vectors of the event marked by the blinded observers. For each dive, the same synchronization time offset was applied to the two observers’ vectors. The PB and IP recording system’s time base running slightly faster than the video camera device’s, probably due to a time calibration insufficiently accurate for both devices, time difference was compensated for in the Matlab scripts.

Finally, the events marked by the detection algorithm were compared to the events marked by observer 1 and 2, and the performance of the algorithm evaluated with the same criteria as above.

## 3. Results

### 3.1. Collected Data

A total of 16 dives were recorded and analyzed, totalling 13.9 h and 11081 marked inhalation events. Table 2 gives more details on the conditions for each dive. A total of 15 dives were properly recorded by the video camera device and analyzed by the two blinded observers, for a total of 154 min of video feed analyzed and 2339 marked inhalations by observer 1 and 2334 marked inhalations by observer 2.

### 3.2. Optimization

**Figure 13:**
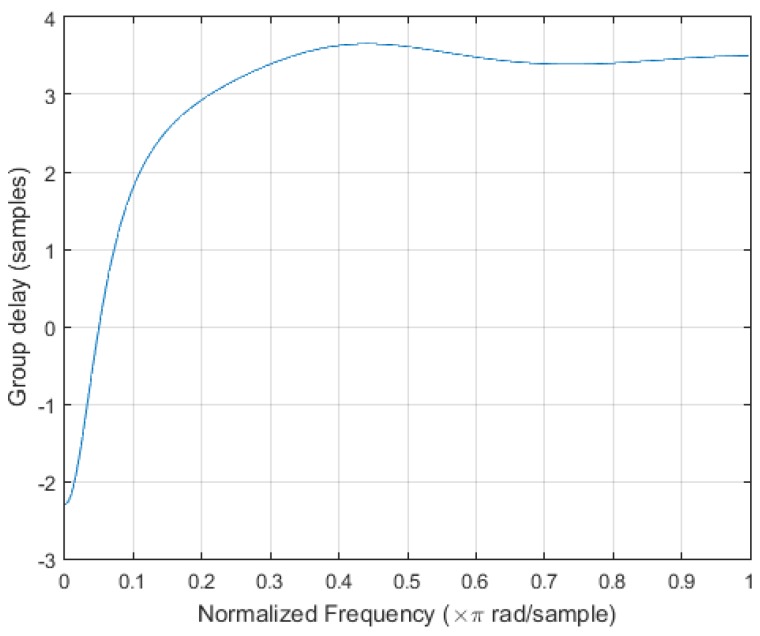
Response delay of the first order Butterworth low-pass filter used in the inhalation detection algorithm.

Optimization of the algorithm parameters based on the collected data and the marked events has given a solution:

(7) $$\left\{\begin{array}{cc} Tr & =0.55\\ Fc & =0.037\\ Diff & =0.3\;\mathrm{bar} \end{array}\right.$$

with a step size *N* of 100 samples (5 s) and a maximum delay of the Butterworth filter of 4 samples (0.2 s) as shown in Figure 13, the delay between actual inhalation and detection by the algorithm will be in the worst case of 5.2 s, meeting the initial requirements of ten seconds maximum.

In order to execute the algorithm while embedded in a microcontroller, at least (M+N) IP sensor measures must be stored in memory. PB compensation will be executed on the fly, removing the need to store PB signal in RAM. Each pressure value is encoded in 20 bits (2.5 bytes). The total RAM required to properly execute the algorithm is therefore: 2.5×(N+M)=2.5×15×20=750 bytes, meeting the initial requirements of 6 kBytes. It is a reasonable assumption that another 50 bytes of RAM will be necessary for the proper execution of the algorithm, to store various variables and indexes.

### 3.3. Algorithm Results

Table 2 lists the number of TP, FP and FN and the values of the sensitivity and the ratio *R* for each dive. The arithmetic mean sensitivity, time-mean sensitivity, the total sensitivity and the time-event weight sensitivity (as defined in [21]) are all equal to 97.5% when rounded to the first decimal.

### 3.4. Validation

The vectors of events marked by the observers based on the camera video recordings were compared to one another to evaluate the reproducibility of the process of identification of breathing events by analysis of the video recordings alone. The results are displayed in Table 3. The total process over the 15 dives had a sensitivity of 97.6% and ratio *R* of 2.4% for the data of observer 2 against observer 1, and a sensitivity of 94.5% and ratio *R* of 5.7% for the data of observer 1 against observer 2.

Synchronization of the vectors marked by the video observers with the vector of detected events by the algorithm being a tedious task, only one dive, dive 16, was synchronized and compared to the results of the algorithm. Over the 10.4 min of dive analyzed, the algorithm presented a sensitivity of 99.0% and a ratio R of 20.4% against the analysis of observer 1, and a sensitivity of 99.0% and a ratio R of 21.6% against the analysis of observer 2.

## 4. Discussion

In this study, the IP of the regulator of scuba divers and the PB signals were acquired in real diving conditions and an inhalation live-detection algorithm using these signals was designed and implemented with Matlab. The algorithm’s variables were optimized to obtain a high sensitivity and a low number of FP based on the collected dataset. On the dataset, the sensitivity of the algorithm was 97.5% and the total number of FP was 275 for 13.9 h of recording. The algorithm is expected to use 800 bytes of RAM and to present a maximum delay of 5.2 s.

To the best of our knowledge, this study is unique by using the IP signal to monitor the breathing of the scuba diver.

### 4.1. Limitations

The initial requirement that the algorithm must be executed on a low-power microcontroller with a maximum frequency of 25 MHz remains untested. Any estimation of execution cycles and time will be too approximate to quantify, and will depend partly on the compiler’s optimization. However, since the global operation of the algorithm remains simple and involving integers, it is expected that it should execute under a second to analyze a full window. The low-pass filter, involving floating point calculation, is expected to be the longest step of the algorithm to execute. Another potential issue is that the environment running on the microcontroller is not multi-threading, which may make the implementation delicate. The risk being that during the analysis of the acquired signal, no more sensor data may be acquired until the analysis has ended.

Sources of error that may have haltered the results have been identified. (1) Human error during the manual marking of the inhalation events may be the most significant. Since a large number of events have been marked manually, it is a reasonable assumption that a certain proportion of the events have been skipped, marked twice or mistakenly marked. It must be noted that verifications have been applied to remove events marked twice, but these verifications have limitations on their own; (2) The BC is an indispensable piece of equipment of the scuba diver, enabling balancing buoyancy underwater. Air is injected into the BC from the IP line. When injecting air into the BC, the IP is altered in a very similar manner to the diver’s inhalation event. It is often difficult to separate BC inflation from inhalation when manually marking the inhalation events.

Although the developed algorithm aims at being applicable to all scuba diving activities, this study has limitations: (1) all subjects were experimented scuba divers. It is a possibility that the breathing habits of inexperienced divers are different and affect the IP signal, and therefore affect the global performance of the algorithm; (2) All dives were performed with a maximum depth of 7 m, while the device was designed to perform up to 100 m. It is again a possibility that the IP signal is altered in deeper dives, affecting the algorithm’s performance.

Although it was shown that validation through video observation for identification of inhalation events was a reproducible process, the algorithm was compared to only one dive. Additionally, the act of synchronizing the video marked events with the algorithm’s detected event ensures one biased TP as the first event and one TP as the last event.

### 4.2. Applications

While a remote passive or active acoustic monitoring of scuba divers solution presents the advantage of requiring no device be embedded on the SCUBA divers, there has been no study on the sensitivity of such system applied to a group of divers and the effects on non-breathing event detection mechanism. It should be expected that the system, while able to detect the presence and location of the group of divers, may be unable to isolate each diver’s breathing sound to identify a non-breathing event, as the sound of the other divers would cover the event. It is therefore required that a device such as the system presented in this study be embedded on the diver’s equipment.

The system and algorithm presented in this study are a technology which aims at being implemented in existing or larger systems, such as dive computers. It only requires two pressure sensors (one of which is typically already available in every dive computer) and a microprocessor. The light-weight algorithm makes it easy to implement in various systems.

Additionally, the system can be used as a method of continuous monitoring of the scuba first stage regulator performance. Indeed, the IP is expected to be about 9.6 bar above the PB (the value changes with the regulator model and manufacturer). A higher IP may cause the second stage of the regulator to not withstand the excessive pressure and let gas flow out. This is called free flow, and may cause the tank to empty very quickly while the diver is underwater, leading to shortage of breathable gas. On the other hand, a lower IP may require additional effort from the diver to inhale, causing extra fatigue and increasing the risk of decompression sickness. In the worst case, the low IP will make it impossible to inhale breathable gas from the second stage regulator. The presence of the PB and IP sensors in the systems enables a continuous monitoring of the IP relatively to PB, which should enable early detection of the regulator going out of specification range which could potentially cause a dive accident.

From the detection of inhalation events can be deduced the respiratory rate, which can be used to identify novice’s irregular breathing, hyperventilation and skip-breathing, helping prevention of breathing related dive accidents.

Traditional communication methods used on surface such as radio frequency (RF) must be adjusted when applied to the underwater, due to the physical properties of the medium [22]. Optical [23,24], RF [22], magnetic induction [23], and acoustic [25] communication methods and combinations [26,27] have been developed for underwater applications. Several original equipment manufacturer (OEM) acoustic modems are commercially available, such as the S2C OEM Modem (EvoLogics GmbH, Berlin, Germany) or the SAM-1 (Desert Star Systems LLC, Monterey Bay, CA, USA), offering communication channels to any underwater electronic device. Combined with an underwater communication system, the solution presented in this study would enable automatic reporting to the rescue authorities located nearby on the surface or underwater. If bridged to an internet connection on the surface, the reports could be centralized to the Life Support System platform proposed in [28] or to a platform such as DAN Emergency Hotline, which provides 24 h a day medical assistance and support to handle diving emergencies such as decompression sickness, arterial gas embolism, pulmonary barotrauma or other serious diving-related injuries. Underwater acoustic communication having limited data rates, and RF, magnetic induction and optical communication having limited ranges [23], it remains essential that the algorithm identifying emergency situations such as a non-breathing diver be embedded on the device equipping the diver.

The technique described in this study is subject to a Turkish patent application [29].

## 5. Conclusions

The system and the algorithm presented in this study have demonstrated they enable a new method of continuous monitoring of the breathing of a scuba diver in activity underwater. The algorithm has shown a global sensitivity as high as 97.5% and a low number of 275 false positive inhalation events detected against the 11081 marked inhalation events. It has been determined that such algorithm requires only 800 bytes of RAM to execute, leaving a tiny footprint to even the smallest modern, non-DSP microprocessors and microcontrollers.

Our next aims are to implement and embed the algorithm in the MSP430F5529 microcontroller of the recording system in order to confirm the performance of the solution proposed in this study. A protocol shall be established, similar to the present study, to quantify the sensitivity and number of FP of the recording system with the embedded algorithm.

## Figures and Tables

**Table 1:** Specifications of the recording system.

Specification	Value	Unit
PB Sensing Resolution	5.4	mbar
Absolute Accuracy	±100	mbar
Sampling Rate	1	Hz
IP Sensing Resolution	0.5	mbar
Absolute Accuracy	±100	mbar
Sampling Rate	20	Hz
Maximum Depth	100	m

**Table 2:** Specifications of recorded dives and algorithm performance per dive.

Dive	Diver	Tank	Max. Depth	Duration	Ni ${}^{1}$	TP ${}^{2}$	FP ${}^{3}$	FN ${}^{4}$	Sensitivity	R
Dive 1	Diver 1	Tank 1	6.6 m	50 min	505	494	5	11	97.8%	1.0%
Dive 2	Diver 1	Tank 1	6.3 m	57 min	461	437	62	24	94.8%	13.4%
Dive 3	Diver 2	Tank 2	4.5 m	49 min	881	854	30	27	96.9%	3.4%
Dive 4	Diver 2	Tank 2	4.5 m	49 min	696	691	4	5	99.3%	0.6%
Dive 5	Diver 3	Tank 1	6.4 m	47 min	673	640	19	33	95.1%	2.8%
Dive 6	Diver 3	Tank 1	6.8 m	49 min	541	527	23	14	97.4%	4.3%
Dive 7	Diver 4	Tank 1	6.5 m	43 min	698	653	18	45	93.6%	2.6%
Dive 8	Diver 4	Tank 1	6.8 m	55 min	718	706	3	12	98.3%	0.4%
Dive 9	Diver 5	Tank 1	5.9 m	51 min	770	768	3	2	99.7%	0.4%
Dive 10	Diver 5	Tank 1	6.7 m	55 min	729	724	5	5	99.3%	0.7%
Dive 11	Diver 6	Tank 1	6.8 m	64 min	908	866	15	42	95.4%	1.7%
Dive 12	Diver 6	Tank 1	5.8 m	69 min	968	948	10	20	97.9%	1.0%
Dive 13	Diver 7	Tank 1	5.7 m	47 min	879	861	3	18	98.0%	0.3%
Dive 14	Diver 7	Tank 1	5.4 m	50 min	616	610	25	6	99.0%	4.1%
Dive 15	Diver 8	Tank 2	4.7 m	46 min	592	580	16	12	98.0%	2.7%
Dive 16	Diver 8	Tank 1	6.9 m	50 min	446	443	34	3	99.3%	7.6%

${}^{1}$ Marked events; ${}^{2}$ True positive; ${}^{3}$ False positive; ${}^{4}$ False negative.

**Table 3:** Results of video analysis and comparison of observer 1 data vs. observer 2.

Dive	Duration ${}^{1}$	Observer 1 vs. Obs. 2			Observer 2 vs. Obs. 1		
		Ni ${}^{2}$	Sensitivity	R	Ni ${}^{2}$	Sensitivity	R
Dive 1	10.6 min	129	93.0%	7.8%	130	96.9%	2.3%
Dive 2	10.6 min	108	88.0%	14.8%	111	97.3%	0.9%
Dive 3	10.4 min	194	94.5%	4.6%	193	97.9%	2.6%
Dive 4	10.2 min	174	94.3%	6.9%	176	98.3%	0.6%
Dive 5	10.8 min	172	98.3%	2.3%	173	99.4%	0.6%
Dive 6	10.3 min	170	97.1%	2.4%	169	98.2%	2.4%
Dive 7	8.4 min	150	92.7%	6.0%	148	97.3%	4.1%
Dive 8	10.7 min	161	87.0%	13.0%	161	95.7%	4.3%
Dive 9	10.1 min	181	95.6%	4.4%	181	99.4%	0.6%
Dive 10	10.2 min	163	95.7%	3.7%	162	99.4%	1.2%
Dive 11	10.3 min	136	91.2%	5.9%	132	91.7%	11.3%
Dive 13	10.4 min	214	95.3%	7.5%	220	95.4%	1.8%
Dive 14	10.2 min	144	97.9%	1.4%	144	100.0%	0.7%
Dive 15	10.6 min	136	97.1%	3.7%	137	97.1%	2.2%
Dive 16	10.4 min	102	98.0%	2.9%	103	99.0%	1.0%

${}^{1}$ Duration of the analyzed video; ${}^{2}$ Marked events.

## Abbreviations

The following abbreviations are used in this manuscript:

BC	Buoyancy compensator, Buoyancy compensator device or Buoyancy control device
DAN	Divers Alert Network
DSP	Digital signal processor or digital signal processing
ECG	Electrocardiogram
FN	False negative event detected
FP	False positive event detected
IP	Intermediate pressure
OEM	Original equipment manufacturer
PB	Ambient pressure (absolute)
PC	Personal computer
RAM	Random-access memory
RF	Radio frequency
SD	Standard deviation
SCUBA	Self-contained underwater breathing apparatus
TN	True negative event detected
TP	True positive event detected
